# *What does it mean?* Translating anatomical language to engage public audiences

**DOI:** 10.1002/ase.70007

**Published:** 2025-02-16

**Authors:** Kat A. Sanders, Adam M. Taylor

**Affiliations:** ^1^ School of Medical Sciences, Faculty of Medicine & Health University of Sydney Camperdown New South Wales Australia; ^2^ Centre for Anatomical and Human Sciences, Hull York Medical School University of Hull Kingston Upon Hull UK; ^3^ Lancaster Medical School, Faculty of Health and Medicine Lancaster University Lancaster UK

**Keywords:** anatomical language, eponyms, public engagement, science communication, science outreach education

## Abstract

The language of anatomy, with its roots in Ancient Greek and Roman languages, is complex and unfamiliar to many. Its complexity creates a significant barrier to public knowledge and understanding of anatomy—many members of the public find themselves asking “what does it mean?”, and this can manifest as poor health literacy and outcomes. To address this, anatomists who interact with the public should be able to translate anatomical language and support the development of individuals' foundational understanding of why structures are named the way they are. In this review, language is categorized by themes that inform the naming of anatomical structures, such as function, location, and appearance, and paired with pedagogical approaches informed by education and public engagement research to underpin effective communication that demystifies the language of anatomy for public audiences. Drawing on pre‐existing sometimes unrelated knowledge, gamification and quizzes can make language more accessible and recognizable. Humorous etymological insights into the origins of anatomical terms can build rapport between anatomist and public audience and normalize discussions about sensitive topics, such as genitalia, in an inclusive manner. Finally, eponyms, while contentious due to their lack of diversity and ethical concerns, can serve as narrative subjects to start discussions that address anatomy's relevance to wider socio‐political and bioethical discourse. Ultimately, by deploying established science communication tools when translating anatomical language, anatomists can deliver effective public engagement that cultivates ongoing curiosity in anatomy, its language, and more broadly health.

## THE IMPORTANCE AND CHALLENGES FOR PUBLIC AWARENESS OF ANATOMICAL LANGUAGE

Anatomy is the cornerstone of the medical sciences; the language of anatomy has its roots in Ancient Greek and Roman languages and cultures.^1^ In these ancient times, all sciences were limited to predominantly descriptive terms for things, one that is better known is that of astronomy and the star signs. The literal translation of Gemini as the twins forms part of a much older example but demonstrates the same principles that anatomists observe in the terminology they see daily. As the fascination with the body grew, dissection of bodies to understand normal and diseased human structure became commonplace.^2^ Unfortunately, many of their functional descriptions were wrong because they did not have the scientific techniques available today to understand what was happening in terms of health and disease, or even normal function. Examples of errors postulated by some of the most famous anatomists such as Galen include failing to understand the differences between systemic and pulmonary circulations, the origin and destination of arterial and venous blood, as well as recognizing the lung as the organ that facilitates gas exchange.^3^ Things were seen on a very macroscopic level, with microscopic and molecular techniques hundreds and thousands of years away, respectively. Led by Sylvius and Bauhin in the 16th and 17th centuries, anatomists set about naming anatomical structures^4^ using language from the Ancient Greeks and Romans. However, they were still limited to describing structures based on their appearance and postulations about their function.

Scientific advancements have helped us better understand the function of structures and the pathogenesis of many diseases that were first described hundreds of years ago. Despite correcting historical assumptions and inaccuracies, we continue to use many of the anatomical terms published during the late Renaissance. In the recent years, the Federative International Programme for Anatomical Terminology created the *Terminologia Anatomica* to help establish a global common nomenclature for anatomical structures, now in its second edition.^5^ This anatomical language is similarly widely used in medicine, as well as other subjects as a standard means to communicate and describe structures, locations, their relations, and pathologies. One of the challenges around understanding these ancient languages and their influence on the languages of anatomy and medicine is that they are seldom taught in schools. In the early 20th century, very little had changed from Victorian times, and opportunities to learn these languages in the United Kingdom was limited to those who were deemed to be suitably academically gifted, such as Grammar school pupils.^6^ In the 1960s and 1970s, Latin began to appear on comprehensive school subject offerings, with hundreds providing it as an educational option.^7^ However, lessons on these languages have recently declined; a British Council survey in 2021 found that only 2.7% of public schools, compared to 49% of independent schools offered lessons in Latin.^8^

Given this decline, a key challenge related to the comprehensibility of anatomical language is that even students and professionals who are learning anatomy may be unaware of the origins or understanding of what anatomical terms mean. With the growing accessibility of medical records to patients, these terms and references are now widely available to patients, which present the issue of lack of knowledge or understanding about their meaning(s).^9^ This can create confusion and worry for patients and potentially increase the burden on healthcare services, as patients try to gain follow‐up appointments to find out what things mean or how they may need further management. While technical medical terminology is commonplace, if healthcare professionals are not able to effectively translate this language, it creates issues when communicating clearly with their patients.^10^, ^11^, ^12^

## ANATOMICAL KNOWLEDGE IN THE PUBLIC AND ITS POTENTIAL VALUE

Adult knowledge about anatomical and health terms is varied; in North America, 40% of adults have marginal, and 22% have inadequate health literacy levels which was linked with hospital readmissions.^13^ Those with poor health literacy have difficulty understanding prescription labels, participating in medical decisions, following medical recommendations, and attending their follow‐up appointments.^13^, ^14^, ^15^ A similar study demonstrates that males, younger individuals, immigrants, those with basic levels of education, a wage below the national average, or receiving state benefits were all associated with poor health literacy. Those individuals with inadequate health literacy levels were more likely to revisit emergency departments, increasing demand on healthcare resources and provision as well as potentially negatively impacting their treatment and recovery.^16^

Specifically in anatomy, colleagues have shown that there is a global lack of public knowledge of anatomical structures and their location.^10^, ^11^ It could be assumed that if they cannot identify the location of structures, they may not be able to understand their function or other affiliated knowledge. Indeed, it seems that it is the complexity of anatomical language that exacerbates the difficulty in identifying structures. Evidence to support this is seen from the Anatomy Nights study looking at brain knowledge of international audiences attending anatomy outreach events.^17^ Participants at Anatomy Nights were asked to identify a series of structures on an image of the brain. Structures that had non‐science specific terms in their names, such as “brain stem” and “frontal lobe,” scored so highly in pre‐event tests that there was little scope for improvement in post‐event tests. The names of these anatomical structures reflect the normal definitions of these words: the brain stem descends from under the brain, like a plant's stem is under the flower; the frontal lobe is at the front of the brain. While it cannot be stated with certainty that it is the public's familiarity with the words rather than already knowing what these structures are, these were the only questions without anatomical jargon in their name assessed in this study. In contrast, terms with more scientific names (e.g., occipital and parietal lobes, cerebellum) scored poorly in the pre‐event tests, with improvement after the Anatomy Nights event.^17^

The issue of the complexity of anatomical language is not a new one and with a multitude of exposure to anatomical (and medical) terms throughout each individual's life, there is a growing need to ensure that the public can better understand what they are hearing and reading. This presents an opportunity and a responsibility for anatomists to incorporate an understanding of this language into engagement and learning opportunities for the public in the hope that they can learn and make (more) informed decisions, particularly when this information relates to their health or that of their family and friends.

## BREAKING DOWN ANATOMICAL LANGUAGE FOR PUBLIC AUDIENCES

### Words and their meaning

Anatomists who engage with public audiences need to be able to demystify the language of anatomy. To do this, categorizing anatomical names into core themes can provide a structure for presenters and audiences alike to follow. Examples of Latin and Greek names of structures are typically found in major categories; function, location, and appearance; these will be explored further.

Those structures named for *function* include muscles such as “flexor,” “extensor,” “levator,” “pronator,” and “tensor.” These muscle descriptors offer clues as to the action of the muscle(s) at joints or in moving bones. Putting these terms into the full context of the muscle enables a greater understanding of the location and function. Indeed, this can be signposted to public audiences, encouraging them to look for familiar words and use these to elucidate the function of a structure. For example, “flex‐,” “digit‐,” and “superficial‐” in *flexor digitorum superficialis* tells you that it is a superficial flexor of the digits.

The structures named for their *location* (Table 1), provide a useful description once translated. Similarly, names that refer to attachment sites (e.g., sternocleidomastoid) allow the anatomist to (re)introduce palpable skeletal features (sternum, clavicle (cleido), mastoid process) that audiences can locate on their own bodies, and by understanding attachment, audiences can develop an understanding (rather than simple knowledge) of the function of the muscle.

**TABLE 1:** Examples of anatomical terms related to location of a structure, and how these are represented in anatomical names.

Location	Translation	Example
Brachial	Brachium (arm)	Brachial artery
Dorsal	Upper surface (of an organism)	Dorsal
Orbicularis	Orbits around (an opening)	Orbicularis oris (muscle)
Sternothyroid	Sternum to thyroid	Sternothyroid (muscle)
Parietal	Body wall	Parietal pleura
Visceral	Organ	Visceral pleura

Next, there are structures that are described for their *appearance* (Table 2). Some are obvious to those without exposure to anatomical or medical language, such as longus, magnus, and rhomboid all falling into this category, descriptors of long, large, and rhomboid in shape.^18^ While other examples may require further explanation (Table 2).

**TABLE 2:** Examples of anatomical terms related to the appearance of a structure, and how these are represented in anatomical names.

Appearance	Translation	Example
Brevis	Short	Adductor brevis
Quadrate	Four‐sided	Quadratus lumborum
Trapezius	Trapezoid	Trapezius
Fornix	Arch or vault	Vaginal fornix
Nigra	Black	Substantia Nigra
Lunate & Meniscus	Crescent moon shaped	Lunate bone (wrist)
Fungiform	Mushroom	Fungiform papillae (tongue)
Sesamoid	Sesame‐seed like	Sesamoid (bone)
Chorda	Strings (of an instrument)	Chorda Tympani (nerve)
Tympanum	Drum	Tympanic membrane (ear)
Corona	Crown	Coronary artery
Annulus	Ring	Annulus Fibrosus
Mitral	Bishop's Mitre (hat)	Mitral valve

### Games and competition—Making it fun

Rather than trying to rote learn these in a list or medical context, anatomists have moved to try and engage learners with fun and competition, gamifying anatomy for public and university audiences alike. This progresses from one of the most popular cards games in the recent years “Organ Attack”.^12^, ^19^ Others have developed their own games to assist with student learning and understanding by bringing their own games forward that combine learning with the competitive nature and fun that reduces the cognitive focus. One such example is Wordotomy which looks at the basis of the anatomical words and what they mean.^20^ Another avenue to incorporate active audience engagement with anatomical language is to interrogate these names through the medium of traditional party games like charades and Pictionary. It has been demonstrated that these games have a positive impact on university students' learning of scientific vocabulary^21^, ^22^ and can easily be applied to a live public audience. Furthermore, quiz type activities are favored when integrated into learning over didactic style lecture delivery.^23^ They enable learners to check their progress and integrate the focus on names of structures and their function in smaller parts, rather than all in one didactic block. Additionally, the value of educational games has been demonstrated in children who are attending asthma clinics, with positive knowledge gain in 84% of outcome measures assessed, which in turn can reduce morbidity risk from understanding correct condition management.^24^ Most recent developments are also seeing the introduction of artificial intelligence into anatomical education through gamification. These studies reinforce the benefits of gamification on anatomical learning as well as using the virtual assistant to direct students to areas where further student learning is required.^25^, ^26^ Educational value is also significantly improved, along with student feedback when outcomes and student feedback is incorporated into subsequent learning resources.

When considering anatomical (and medical) language as “a new language” which given its roots in Latin and Greek seems reasonable, you can view a student's learning journey as one of learning a new language, given the hundreds, if not thousands of anatomical and medical terms they will learnt over the course of their studies and continue to do so as part of their ongoing training.^27^ Apps which teach new languages, such as Duolingo, have shown positive effects on language learning and potential in health education.^28^

This gives clear evidence and hope that integrating games and quizzes into public engagement will have a positive impact on language knowledge, in the same way that it is proving to be a positive resource in formal and informal anatomy learning fora.

### The power of a good story

Narratives are a valuable and versatile addition to any science communicator's toolkit. Dudley et al. found that narrative was more effective than didactic communication strategy at conveying scientific and health content to public audiences in fifty of seventy‐eight articles (64%) reviewed.^29^ Additionally, audiences receiving information in narrative form are more likely to remember details,^30^ so useful and relevant anatomical knowledge can be woven into a story of how an anatomical structure received its name. It is also important to highlight that narratives have been found to be effective on audiences with limited health literacy,^31^ thus, anatomists should focus particularly on incorporating various elements of narrative story telling into their outreach activities.^32^

A fun example of etymological narrative can be found with the sartorius muscle, whose name belongs (by virtue of a good story) in the *function* category of naming. The sartorius muscle crosses the hip and knee joints, acting to flex both joints and laterally rotating the hip. The synergistic combination of these actions is crucial to assuming a cross‐legged position; a position tailors typically assume when sewing in the canvas of a bespoke suit. In Latin, *Sartor* means tailor and so the muscle's name is linked to its applied function. This etymology has also been applied to the English word “Sartorial”; an adjective that relates to clothing and is generally used to denote that someone dresses well (i.e., their clothes are tailor‐made). These elements related to the sartorius muscle can be combined to produce a story underlying the muscle's etymology while also capturing its function in a memorable narrative. Whether within a structure's etymological origins, or otherwise, there is no shortage of opportunities within the vast field of anatomy to craft an education tale that remains with the audience.

### The taboo subjects—Using humor to build rapport

Returning to focus on naming anatomical structures for their visual similarity to other things, it is interesting to observe that there is an abundance of structures named for their resemblance to breasts or nipples. In some cases, the rationale for such nomenclature is readily apparent, as exemplified by the mammillary bodies on the base of the brain (Latin *mamila* for little breast). However, in other cases, the justification for such terminology requires a more substantial leap of imagination to see the resemblance and to believe that there were no better options. Examples of this include the mastoid process (Greek *mastoeides* for breast like) and the papillary muscles of the heart (Latin *papilla* for nipple).

From a public engagement perspective, this wealth of structures carrying names related to breasts and nipples presents a valuable two‐step opportunity to meaningfully connect with audiences. First, and most simply, by leaning into the juvenile humor surrounding the overactive imaginations of the anatomists who established anatomical nomenclature in the 16th and 17th centuries. When done respectfully, humor directly associated with scientific content can be highly effective at maintaining audience engagement.^33^, ^34^ The second and most important step of this engagement opportunity hinges on this use of humor to build rapport between presenter and audience. Using humor has been shown to increase audiences' help‐seeking behavior relating to stigmatized symptoms and health conditions,^35^ and once rapport is established, audiences may be more receptive to discussing topics considered sensitive or taboo. It is well recognized that the public can be reticent to engage with health screening campaigns or reporting symptoms for conditions related to genitalia.^36^, ^37^, ^38^ By openly acknowledging and speaking about genitalia in anatomy outreach, members of the audience may then feel more open to speaking to their doctors about issues and be able to use and understand the associated anatomical language. They might also directly engage in dialogue with the presenter, who could encourage them to consult a health professional. A key goal of anatomical public engagement is to build public awareness of the body, and in turn influence audience behavior to improve health outcomes. This is an example of how an anatomist can use humor to establish a relationship with a public audience by drawing on the etymology of anatomical terms, and leveraging this to normalize important conversations that are too often avoided.

It should be noted that any discussion related to the anatomy of genitals must be done in an inclusive manner. It is counterproductive to reduce the stigma related to conditions considered sensitive, and at the same time, disaffect individuals who may be intersex, transgender, or gender diverse. Using person‐first, anatomy‐based language (e.g., people with a prostate or “the [specific structure]”) rather than binary, sex‐based language (e.g., in “males”) allows everyone to discuss anatomy inclusively.^9^ This use of inclusive language has been successfully demonstrated in university learning environments^9^, ^39^, ^40^, ^41^ and it should be no different when anatomists communicate with public audiences.

### Eponyms—Out with the old?

Finally, no discussion on anatomical language would be complete without exploring *eponyms*. Eponyms are identifying terms usually derived from the name of a person. Commonly used examples in anatomy include the Achilles tendon, Circle of Willis, Fallopian tube, and McBurney's point. Unlike the aforementioned naming conventions based on Greek and Latin words, eponymous terms do not provide any information about the structure in question (i.e., function, location, or appearance). Furthermore, all anatomical structures with eponymous names also have formal, internationally agreed upon, descriptive names in the Terminologia Anatomica.^5^, ^42^ This duplication of names for a single structure inherently confuses the already daunting language of anatomy. This is just one of the issues that contributes to the ongoing debate within the anatomy discipline regarding the continued use of eponymous terms for anatomical structures.^43^, ^44^, ^45^, ^46^, ^47^, ^48^ This article does not intend to advance this debate in either direction. Rather than asking *“should* eponyms appear in anatomical public engagement?” this article instead acknowledges that the presence of anatomical eponyms persists, and therefore aims to provide practical guidance for when and how to use eponyms appropriately in anatomy public engagement.

An important reason to use an eponym is if that name is publicly well‐known. From our experiences, Fallopian tube (rather than uterine tube) is often recognized by audiences; though some audience members can be surprised to learn that this is an eponymous term (for Gabriele Falloppio). It is incumbent on anatomists who engage the public to meet audiences where they are, and to bridge the gap created by anatomical jargon. Therefore, where there is already public awareness of an eponymous name (even if they are not aware it is an eponym), this can be used as a foundation to introduce the *Terminologia Anatomica* name and grow health literacy.

Anatomical eponyms may also have value as a marker of our field's history which can be communicated as a narrative. When in vogue, eponyms were used to convey status within the field; for example, anatomists claiming discovery of a structure by assigning it their name, or the name of another person to acknowledge their scientific contribution.^49^ While eponyms do not always accurately recognize this contribution to knowledge (as discoveries are often the work of teams, rather than individuals), the background details of named individuals lend the science communicator a ready‐made story in the form of biography.^50^ As described earlier, narratives are a powerful communication tool that should not be underestimated. However, caution should be taken when using eponyms as a basis for narrative, as there are profound ethical concerns about the actions or beliefs of some persons who have lent their name to anatomical structures.^51^ This is not to say they should not be discussed, but rather it is imperative that anatomists are aware of such examples and have the communication skills necessary to discuss them appropriately and sensitively. Organ and Mussell made the case that eponyms can be used to teach bioethics in the anatomy classroom, and this can apply to the public arena as well.^48^ Depending on the focus of the outreach activity, eponyms might be included to call attention to abhorrent transgressions that have underpinned some anatomical knowledge, with an emphasis on the bioethical implications and corrections that the field has undergone since. By openly acknowledging the sinister aspects of anatomy's history, anatomists have the opportunity to demonstrate accountability on the discipline's behalf and take steps to actively (re)gain public trust in anatomy, and medicine, more widely.

Another key concern for the ongoing use of eponyms is the lack of diversity in the exclusive group for whom anatomical structures share a name.^45^ Within the field of medicine specifically, eponymous terms for clinical conditions and procedures continue to be created. Since the turn of the century, the percentage of women in medicine closely matches the proportion of new medical eponyms attributed to women.^52^ However, this gender balance within the medical field is only recent, and the propensity for ascribing eponymous terms in the 18th and 19th centuries means that only 4% of all medical eponyms are attributed to women.^52^ This discrepancy is exacerbated when examining anatomical eponyms. The discovery of new anatomical structures has been exceedingly rare in the past hundred years, and so eponymous terms associated with women and people from minoritized groups are few and far between in anatomy. Indeed, the only structure that comes to mind is the eye's Iris, named for the Greek *mythological* figure. Eponyms in the anatomical context are also uniquely problematic. There are many commonplace eponyms that feature in our everyday language related to objects and concepts (e.g., Caesar salad, degrees Fahrenheit, Pandora's box, etc.) separate from the body. In contrast, giving parts of the body eponymous names implies ownership of the structure. As such, anatomical eponyms are particularly contentious when men's names have been used to identify structures only found in the anatomy of people with a uterus (e.g., Fallopian tube, pouch of Douglas, Bartholin glands).^53^ In this context, eponyms bolster patriarchal and colonial narratives around bodily autonomy, regardless of whether one is aware that the name is eponymous. Indeed, through this lens, anatomists have the opportunity (and responsibility) to demonstrate for public audiences the connections between anatomical language and current socio‐political discourse around gender equality and decolonization.^54^, ^55^ Furthermore, this facilitates a core facet of scientific public engagement; the co‐creation of understanding by experts and the public which has the potential to advance positive changes socially and within the discipline.^56^

Overall, while the use of eponyms remains contentious, they provide valuable opportunities for anatomists to engage with the public in discussions that use anatomical language as a tool with which to interrogate our history to start making sense of the present.

## SUMMARY

With the importance of understanding anatomical language being highlighted through various modalities in this article, it is important to try and begin to incorporate the solutions to problems when working with public audiences of all ages. It is also important that as anatomy educators, we give future anatomists and healthcare professionals the tools to do the same. It is recognized that in health focussed courses anatomical language becomes a familiar medium of communication, but little focus is given to what the words mean. If an understanding of this language was incorporated into the learning, it might make it easier for this to be translated back to patients in their clinical contact. Combining this with a better understanding of basic anatomy for the public/patient side has the potential to yield benefits to patients as well as healthcare systems. Indeed, by having a focus on names and structures, knowledge becomes more accessible to the public, who may pick up and retain small but life changes pieces of information about structures in their own body.

To finish, a key indicator of outreach success is to inspire an ongoing curiosity for the subject in members of the audience. To take their knowledge further, there are pre‐existing, public‐facing, anatomical language resources that anatomists can sign‐post to a curious audience including books and games.^18^, ^20^, ^57^ Whether delivering a talk specifically on anatomical terminology or breaking down anatomy jargon as part of a larger activity, do consider wielding powerful engagement tools such as games, humor, and narratives; all of which have the potential to not only deliver information in a way more likely to be retained but also strengthen your relationship with the audience. Building on this, translating anatomical terminology related to sensitive topics and eponyms is an invaluable opportunity to demonstrate the links between anatomy and wider socio‐political and bioethical discourse, setting the stage for co‐creation of understanding and strengthening the links with our wider communities.

## AUTHOR CONTRIBUTIONS

**Kat A. Sanders:** Conceptualization; methodology; writing – original draft; writing – review and editing. **Adam M. Taylor:** Conceptualization; methodology; writing – original draft; writing – review and editing.
